# Translating Interdisciplinary Research on Language Learning into Identifying Specific Learning Disabilities in Verbally Gifted and Average Children and Youth

**DOI:** 10.4236/jbbs.2017.76017

**Published:** 2017-06-16

**Authors:** Ruby Dawn Lyman, Elizabeth Sanders, Robert D. Abbott, Virginia W. Berninger

**Affiliations:** 1 Learning Sciences and Human Development, University of Washington, Seattle, WA, USA; 2 Quantitative Methods and Measurement, University of Washington, Seattle, WA, USA

**Keywords:** Defining Specific Learning Disabilities (SLDs), Diagnosing Specific Learning Disabilities in Written Language (SLDs-WL), Verbal Giftedness, Multi-Component Working Memory Endophenotypes, Language Learning Mechanism, Translation Science for Diagnosis of SLDs

## Abstract

The current research was grounded in prior interdisciplinary research that showed cognitive ability (verbal ability for translating cognitions into oral language) and multiple-working memory endophenotypes (behavioral markers of genetic or brain bases of language learning) predict reading and writing achievement in students with and without specific learning disabilities in written language (SLDs-WL). Results largely replicated prior findings that verbally gifted with dyslexia score higher on reading and writing achievement than those with average verbal ability but not on endophenotypes. The current study extended that research by comparing those with and without SLDs-WL with assessed verbal ability held constant. The verbally gifted without SLDs-WL (*n* = 14) scored higher than the verbally gifted with SLDs-WL (*n* = 27) on six language skills (oral sentence construction, best and fastest handwriting in copying, single real word oral reading accuracy, oral pseudoword reading accuracy and rate) and four endophenotypes (orthographic and morphological coding, orthographic loop, and switching attention). The verbally average without SLDs-WL (*n* = 6) scored higher than the verbally average with SLDs-WL (*n* = 22) on four language skills (best and fastest handwriting in copying, oral pseudoword reading accuracy and rate) and two endophenotypes (orthographic coding and orthographic loop). Implications of results for translating interdisciplinary research into flexible definitions for assessment and instruction to serve students with varying verbal abilities and language learning and endophenotype profiles are discussed along with directions for future research.

## 1. Introduction: Defining Specific Learning Disabilities and Giftedness

Defining learning disabilities has been controversial in the United States both legally for qualifying students for special education services and scientifically for research purposes; and the controversies are far from resolved [1] [2]. Approaches that have been used historically in both educational practices in schools and diagnostic practices in clinical settings and in research include IQ-achievement discrepancy (which poses special challenges for those who are gifted and learning disabled), response to intervention (RTI), and pattern of strengths and weaknesses.

### 1.1. Discrepancy between Ability and Achievement

The discrepancy approach has an inherent definitional limitation in its use of the term intelligence quotient (IQ). For over five decades cognitive ability tests have not used quotients based on dividing mental age scores on tests by chronological age and multiplying the result by 100 [3]. Wechsler replaced the intelligence quotients with standard scores, which unlike quotients measure relative standing within a continuous normal curve distribution and permit comparison of an assessed child with age or grade peers at a given point in development and the same child with herself or himself across age or grade. However, even though standard scores (raw scores transformed based on national norms for age or grade) allow interpretation based on the normal curve, Wechsler continued to call the scores IQs. Of note, the publishers of the *Wechsler Intelligence Scale for Children* did not. Recent versions generate a domain-general score (Full Scale Score) and four domain-specific scores (Index Scores): Verbal Comprehension Index (VCI), Perceptual Organization Index (POI), Processing Speed Index (PSI), and Working Memory Index (WMI).

The discrepancy approach also has limitations due to failure to identify the exact amount of discrepancy between cognitive ability and achievement that alone defines a specific learning disability (SLD) and its lack of sensitivity to certain SLDs, for example, those involving problems in oral language learning that lower performance on tests that assess cognition with oral responses [4]. The discrepancy approach may also fail to identify SLDs in individuals who are twice exceptional: their giftedness may compensate for underlying SLDs so their achievement falls in the average range, and their SLDs may mask their giftedness and potential for higher levels of achievement [5] [6].

### 1.2. Twice Exceptional

How giftedness is defined in those who are twice exceptional—both intellectually talented and learning disabled—has also been a source of controversy. Approaches have included (a) domain-general and domain-specific, and (b) fluid intelligence based on brain function and crystallized intelligence based on environmental interaction [7]. Some approaches use verbal measures of cognition, whereas others use nonverbal measures of cognition such as the Raven’s Progressive Matrices and the Naglieri Nonverbal Ability Test (NNAT) [8] [9]. Although cognitive tests are used the most for gifted identification [10] [11], multi-modal assessment is an alternative approach [12]: cognitive tests for initial screening followed by assessments of creativity, divergent thinking, task commitment, interests, and other aspects of giftedness, such as the social world of being intellectually gifted [13]. Sternberg’s Wisdom Intelligence Creativity Synthesized (WICS) model conceptualized giftedness as a mixture of intelligence, and wisdom, and assumes that gifted individuals are not equally strong in all areas, but are able to use their strengths to compensate for their relative weaknesses [7]. Yet another approach is teacher nomination based on observation of a child at school, but teacher nomination for gifted assessment or gifted programs has been found to be limited by inequalities related to gender [14] and race [15]. McBee emphasized that teacher education should prepare teachers to identify gifted characteristics across these diverse groups.

### 1.3. Response to Intervention

The response to intervention (RTI) three-tier model, which was introduced to replace the “wait to fail” model (in which a student must reach a certain size of discrepancy before qualifying for specialized instruction) [16], also has its limitations. Although it is always best educational practice to monitor an individual student’s response to instruction, if the same intervention is used with all students at Tier 1 and a student does not respond to it, it is not clear if the lack of response is due to an SLD or to the intervention not being tailored to the student’s learning profile. Without diagnostic assessment it may not be possible to identify the nature of an individual student’s SLD or design appropriate prevention strategies. Also, evidence-based approaches do not exist for how long a student has to show no RTI for there to be evidence of an SLD [17].

### 1.4. Patterns of Strengths and Weaknesses

For this reason and because some students struggle with language learning due to variations in their language and cultural backgrounds [18], many schools are using comprehensive assessment to identify a pattern of strengths and weaknesses in processing skills [19] [20] [21] to design individualized interventions based on identified specific needs and then monitor response to individualized interventions [22]. The current study was designed to introduce yet a fourth approach that identifies patterns of strengths and weaknesses in evidence-based profiles of biologically based levels of language learning that are relevant to the learning of both typical language learners and those with SLDs [23].

## 2. Research Aims

The current study was grounded in interdisciplinary research (genetic, brain, and psychological), and was designed to extend prior studies by addressing two specific aims and their related research questions.

### 2.1. Research Aim 1

The first specific aim was to evaluate whether results of an earlier multi-generational study of dyslexia would replicate. All participants met research criteria for dyslexia but varied as to whether they were verbally gifted (Verbal Comprehension Index in the superior or very superior range) or average verbally (Verbal Comprehension Index in average range). The verbally gifted group with dyslexia tended to show significantly higher reading and writing achievement than the verbally average group with dyslexia, but the two groups did not differ significantly in multi-component working memory endophenotypes (behavioral markers of genetic and brain bases of dyslexia) supporting language learning [24]. The specific research question linked with *research aim* 1 of the current study was whether these prior research results would replicate for Gifted with SLDs in language (twice exceptional) and Average with SLDs in language differing in language achievement but not working memory endophenotypes.

### 2.2. Research Aim 2

The second specific aim was grounded in prior research showing that a measure of verbal reasoning ability (translating cognitions into oral language on the WISC-III, IV, or V) and measures of the multi-component working memory system supporting language learning contributed significant variance to reading and writing achievement in typical language learners in early childhood and middle childhood [25], and in students with persisting SLDs in written language (impaired handwriting, word reading/spelling, and/or sentence reading comprehension/composition) in middle childhood and adolescence [26]. The new question for the present study was whether when range of verbal ability was held constant the language achievement or endophenotypes varied according to the range in which verbal ability for cognitive-linguistic translation fell within the continuous normal distribution of Verbal Comprehension Index standard scores.

For the first research question linked to research aim 2, superior verbal ability was held constant, and groups with and without SLDs-WL were compared. For the second research question linked to research aim 2, average verbal ability was held constant, and groups with and without SLDs-WL were compared. The research questions linked to research aim 2 were whether, when verbal ability range is kept constant (gifted—first research question, or average—second research question), the ability groups with and without SLDs-WL differ in their language learning profiles and/or their working memory endophenotype profiles. That is, a cognitive measure was included but not analyzed for discrepancy from reading or writing achievement. The assessment employed a pattern of strengths and weaknesses approach informed by prior genetics and brain research linked to clinical behavioral measures of gene candidates and brain variables on the same tasks. (We note that results are not reported for response to intervention, RTI.)

### 2.3. Focus on Specific Learning Disabilities-Written Language (SLDs-WL)

In contrast to much research on learning disabilities that has focused only on reading disabilities, *the current study focused on written language learning disabilities*. Prior research had shown that SLDs-WLs can be diagnosed within a cascading levels of language conceptual framework—from subword letter writing, to word reading and spelling, to written (and oral) sentence comprehension and construction [23]. These written language learning disabilities can occur alone or co-occur with others. Epidemiological studies have shown that writing disabilities are the forgotten SLD [27].

## 3. Method

### 3.1. Developmental Profiles

Phone interviews and parent questionnaires were used to assess developmental profiles across the five domains of development (cognitive, language, sensory-motor, social/emotional, and attention/executive function). The goal was to determine that the student did not have a pervasive or specific developmental disability that might explain the struggle in language learning. SLDs-WL should only be diagnosed in individuals who otherwise are typically developing across the domains of development [28]. Both parent ratings on evidence-based assessment tools and parental responses to open-ended questionnaires about medical, developmental, family, and educational history were used to determine (a) whether oral language problems emerged early in development or at transition to school (K-1), and (b) which written language skills were persisting over time despite early intervention in and outside of school.

Part of the developmental profile involved testing and determining if the Verbal Comprehension Index on the Wechsler Scales, which has been shown to be the best predictor of reading and writing achievement in both referred [29] and un-referred [30] samples, fell in the average range (standard score of 90 to 109) or in the superior range or higher (at or above a standard score of 120). This score was interpreted as a cross-domain measure of cognitive-linguistic translation (Niedo, Abbott, & Berninger, 2014) rather than an IQ and not as a solely cognitive measure [25] [31].

### 3.2. Learning Profiles

Diagnostic assessment of learning achievement profiles was also conducted for many reasons. Unfortunately, IDEA 2004 has been interpreted by some to support identifying SLDs by a lack of RTI for at least two years [17]; that is, RTI can be another “wait to fail” model, as the discrepancy model is. The *Diagnostic and Statistical Manual of Mental Disorders, Fourth Edition* [32] acknowledges that early on students with high verbal ability may read at or near grade level and their reading disabilities are not apparent until fourth grade or later [17]. Too many educators may be giving students a twice exceptional label without adequately identifying and addressing areas of intervention individual students may need [33]. Unfortunately, students who are gifted with mild SLDs are less likely to be identified than those with a more severe SLD [34] [35] because they often score within average range on achievement tests, but well below the student’s ability. As a result, the student does not qualify for the services that might help them achieve their potential. Likewise, students who are gifted and learning disabled are less likely to receive accommodations in the classroom that could facilitate their learning [36]. Rarely are teachers trained to recognize the characteristics of co-existing verbal giftedness and SLDs [35] [37].

Specifically, diagnostic assessment of SLDs-WL was conducted to assess achievement for language by ear (listening), mouth (oral expression), eye (reading), and hand (writing) and cascading increasing levels (units) of language within and across these language domains [23]. However, for the current study, to control for too many multiple comparisons, only language achievement measures most relevant to the hallmark deficits in SLDs-WL are reported: handwriting for dysgraphia, word reading/decoding/spelling for dyslexia, and oral comprehension and expression and reading comprehension for oral and written language learning disability (OWL LD).

### 3.3. Endophenotype Profile

In addition to written language achievement, working memory components, which genetics and brain research have shown are involved in language learning [28] [38], were also assessed. These working memory endophenotypes have different brain bases (regions or locations within regions) than do verbal cognitive abilities (e.g., inferior frontal, dorsal lateral prefrontal, superior frontal regions) [38]. Note that the Working Memory Index on the Wechsler Scales does not assess all these working memory components, but there are clinical measures that do. These component working memory endophenotypes include coding (storing and processing) three word forms—phonological [39], orthographic [40] [41], and morphological [42]; two loops—phonological [43] [44] and orthographic [38]; and the executive functions for supervisory attention (focusing and switching) to regulate the multi-component working memory system that supports language learning and use [45] [46] [47]. Learning disabilities are invisible impairments in this multi-component working memory system inside the learner’s mental world [23] [26] [48] and not visible as are auditory or visual sensory disabilities or ambulatory motor disabilities.

### 3.4. Acquisition Procedures

Students in grades 4 to 9 were recruited through flyers distributed to local schools. Interested parents contacted the research team to learn more about the study. If, after a phone screen, it appeared that the student had a history of SLD-WL rather than another disability or condition or was a typical language learner and parent gave consent and the child gave assent, an assessment at the university was scheduled. All procedures had been approved by the Institutional Review Board (IRB) for conduct of research with human participants; and the research team also complied with the ethical standards of the American Psychological Association.

### 3.5. Measures and Diagnostic Profiles

The following measures were given to assign to diagnostic groups:

#### Verbal ability measure.

The *Verbal Comprehension Index* (*VCI*) (test-retest reliability 0.93 to 0.95) of the *Wechsler Intelligence Scale for Children*, 4^*th*^ *Edition* (*WISC-IV*) [49] was used with norm-referenced scores for age with *M* = 100 and *SD* = 15. Those with VCI scores between 90 and 109 in the average range of the normal distribution were assigned to the verbally average group; and those with VCI scores 120 to 129 in the superior range or 130 and above in the very superior range of the normal distribution were assigned to the verbally gifted group. The VCI is based on three tasks: (a) explaining how two concepts are similar, (b) explaining what words mean, and (c) explaining various aspects of the world in which we live. Oral language is required to express cognitive understanding of concepts, words, and knowledge of the world.

#### Learning profile measures.

Achievement was assessed for language by mouth (oral expression), by ear (listening comprehension), by hand (writing), and by eye (reading). The normed measures were standard scores (*M* = 100, *SD* = 15), scaled scores (*M* = 10, *SD* = 3), or a z-scores based on research studies (*M* = 0, *SD* = 1). All can be interpreted in reference to a normal curve of continuously distributed scores.

Two ***oral language*** measures were used. The *Woodcock Johnson III* (*WJ-3*) [50] *Oral Comprehension* test (test-retest reliability 0.88) was given to measure comprehension of heard language (standard scores). When the examiner pauses, students orally supply a word that makes sense in the unfolding heard text. The *Clinical Evaluation of Language Fundamentals* 4 (*CELF*-4) [51] *Formulated Sentences* (test-retest reliability 0.62 to 0.71) was given to assess oral construction of syntax (scaled scores). Sentences students create orally from provided words are scored for syntactic completeness and acceptability.

Two ***writing*** measures were used. The *Detailed Assessment of Speed of Handwriting* (*DASH*) [52] *Copy Best and Copy Fast* tests (interrater reliability 0.99) were given. A sentence with all the letters of the alphabet is copied in one’s best handwriting and then one’s fastest handwriting (scaled scores).

Five ***reading*** measures were used. For *WJ*-3 *Word Identification* [53] (test-retest reliability 0.95) the task is to orally read increasingly difficult real words from a list without the help of context clues. For the *WJ*-3 *Word Attack* [53] (test-retest reliability 0.73 to 0.81) the task is to orally read increasingly difficult words that are pronounceable but have no meaning. For the *Test of Word Reading Efficiency* (*TOWRE*) *Sight Word Efficiency* [54] (test-retest reliability 0.91) the task is to read orally a list of increasingly difficult real words within 45 seconds. For the *TOWRE Pseudoword Efficiency Test* [54] (the test-retest reliability 0.90) the task is to pronounce increasingly difficult words that have no meaning within 45 seconds. For *WJ*-3 *Passage Comprehension* [53] (test-retest reliability 0.85) the task is to supply a word in a blank that makes sense in the unfolding text. All WJ-3 scores and TOWRE scores are standard scores.

#### Component working memory measures of endophenotype profile

For *phonological word form storage and processing*, *the CTOPP Nonword Repetition* [55] test (test-retest reliability 0.70) was given (scaled scores). The task is to repeat orally pseudowords pronounced by the examiner. For *orthographic word form storage and processing*, the *Test of Silent Word Reading Fluency* (*TOSWRF*) [56] (test-retest reliability 0.92) was given (scaled scores). The task is to mark word boundaries in continuous letter strings (without spaces) within the time limit. For *morphological word form storage and processing, Comes From* [42] was given (z-scores). The task is to determine whether the second word in a word pair “comes from” the first word in the word pair. For phonological loop, the Rapid Automatized Naming (RAN) [57] (test-retest reliability 0.90) was given (standard scores). The timed task is to orally name letters in rows. For *orthographic loop*, the *Alphabet Writing* 15 task [40] is to write the alphabet from memory in legible letters in alphabet order as quickly as possible within 15 seconds (interrater reliability 0.97) (z-score). For *focused attention*, the *Delis Kaplan Color Word Form* (*DKEFS*) [45] *Inhibition* test (test-retest reliability 0.62 to 0.76) (scaled scores) was given. This Stroop test requires orally reading color words in ink colors that conflict with the color name of the word. For *switching attention, Rapid Automatic Switching* (*RAS*) [57] (test-retest reliability 0.90) was given (standard scores). The timed task involves orally naming alternating numbers and letters.

### 3.6 Sample Characteristics

Altogether, 69 students in grades 4–9 qualified for the current study: Verbally gifted students with SLDs-WL (*n* = 27; 18 males, 9 females), verbally gifted students without SLDs-WLs (*n* = 14; 9 males, 5 females), verbally average students with SLDs-WL (*n* = 22; 15 males, 7 females), and verbally average students without SLDs-WL (*n* = 6; 3 males, 3 females). All participants had at least one parent with a college degree; racial and ethnic diversity was representative of the region where the research was conducted, and included White (*n* = 50), Asian (*n* = 4), Black (*n* = 1), Hispanic (*n* = 1), East Indian (*n* = 1), and mixed (*n* = 12).

### 3.7. Data Analyses

To address the research questions linked to each of the two research aims, planned two-group *t*-tests were used to compare mean differences on the measures in the learning profiles and the phenotype profiles between two groups: SLDs-WL and typical language learners. Of primary interest was how the two groups might or might not differ as a function of verbal ability (gifted or average). As such, all possible pairwise comparisons among the four groups were not tested—again, only those relevant to the *a priori* research questions linked to research aims to avoid unnecessary Type I error inflation. Means, standard deviations, *t*-test results, and effect sizes (Cohen’s *d*) are reported.

## 4. Results

### 4.1. Research Question for Research Aim 1

See Table 1 for descriptive statistics and t-test results for the first research question. Not surprisingly, given the way the groups were identified, the groups differed significantly in their verbal ability. They also differed in most language achievement skills (both oral language, both handwriting, and three of the five reading—all but accuracy and rate of oral pseudoword reading) but not most endophenotypes (only orthographic coding). Essentially the results of the prior study comparing verbally gifted and verbally average students with dyslexia [24] replicated in this sample that included not only dyslexia but also dysgraphia and OWL LD (multiple persisting SLDs-WL).

### 4.2. Research Question 1 for Research Aim 2

See Table 2 for descriptive statistics and t-test results for both research questions for Research Aim 2. Not surprisingly, given the way the groups were identified, both groups that met inclusion criteria for verbally gifted did not differ significantly on the *WISC-IV Verbal Comprehension Index* (*VCI*). However, the verbally gifted without SLDs-WL scored higher than the verbally gifted with SLDs-WL on six language skills (oral sentence construction, best and fastest handwriting in copying, single real word oral reading accuracy, oral pseudoword reading accuracy and rate) and on four endophenotypes (orthographic and morphological coding, orthographic loop, and switching attention).

### 4.3. Research Question 2 for Research Aim 2

As also shown in Table 2, not surprisingly, given the way the groups were identified, both groups that met the inclusion criteria for verbally average did not differ significantly on the *WISC-IV Verbal Comprehension Index* (*VCI*). However, the verbally average without SLDs-WL scored higher than the verbally average with SLDs-WL on four language skills (best and fastest handwriting in copying, accuracy and rate of oral reading of real words and pseudowords) and two endophenotypes (orthographic coding and orthographic loop).

### 4.4. Comparison of Common and Unique Findings across the Verbally Gifted and Verbally Average Groups (Both Research Questions for Research Aim 2)

Table 3 summarizes the language achievement skills and the endophenotype measures on which the groups did and did not differ. Regardless of verbal ability (gifted or average), groups with SLDs-WL differed from groups without SLDs-WL on orthographic coding, orthographic loop, and best and fastest handwriting. Only the verbally gifted groups with and without SLDs-WL differed on accuracy of oral reading of real words and pseudowords, as well as morphological coding. Further, only the verbally average groups with and without SLDs-WL differed on rate of oral reading of real words and pseudowords.

## 5. Discussion

### 5.1. First Research Question for Research Aim 1

As shown in Table 1, results provide further evidence that giftedness can mask learning disabilities [5] [6]. Academic achievement in language skills may tend to be in the average range in the verbally gifted with SLDs-WL but not the verbally average with SLDs-WL [24]. However, the results also show that those with SLDs-WL, whether verbally gifted or average, do not tend to differ in the endophenotypes, which are non-academic hallmark impairments in specific learning disabilities [24 and current study]. These results are consistent with other evidence that assessment of verbal ability for translation of cognitions into oral language and assessment of working memory endophenotypes are both relevant to diagnosis of SLDs-WL [23] [26].

### 5.2. First Research Question for Research Aim 2

The results of the analyses reported in Table 2 for those who are verbally gifted, but contrast in whether they do or do not have SLDs-WL, extended the prior research by comparing the verbally gifted with and without SLDs. The prior research had not included a control for verbal giftedness without SLDs-WL. The findings showed that, when verbal giftedness was held constant, the groups differed in both language skills and endophenotype measures for SLDs. These findings provide additional evidence that the SLDs-WL in the verbally gifted can be associated with lower language achievement as well as lower scores on the biologically based behavioral markers of SLDs-WL, that is, endophenotypes for the multiple components of working memory supporting language learning.

### 5.3. Second Research Question for Research Aim 2

As the results of the analyses reported in Table 2 show, those who are verbally average, but contrast in whether they do or do not have SLDs-WL, do not differ in verbal ability. The prior research had not included a control for average verbal ability without SLDs-WL. The current findings showed that, when average verbal ability was held constant, the groups differed in both language skills and endophenotype measures for SLDs. These findings provide additional evidence that the biologically based behavioral markers of SLDs-WL for the multiple components of working memory supporting language learning can lower language achievement compared to those who are verbally average without SLDs-WL. For those with average reasoning ability, all these skills involve orthography (written letters or words)—copying written letters in written words in written sentences or translating written real words or pseudowords, which have no associated meaning, into oral pronunciations or writing the alphabet from memory or orthographic coding (detecting written words in letter strings); and five of the six are timed. None of the affected measures involved phonology alone. However, orthographic-phonological correspondences were involved in the reading measures.

### 5.4. Comparing Research Questions 1 and 2 for Research Aim 2

As shown in Table 3, when verbal ability was held constant, those with and without SLDs-WL shared some of the same differences in language skills or working memory endophenotype measures but also unique ones. For both the gifted verbally and average verbally, however, the presence of a diagnosed SLDs-WL was associated with lower orthographic coding and orthographic loop. These findings provide converging evidence that orthographic skills contribute uniquely to reading and writing achievement beyond verbal ability for translating thought into language in students with SLDs-WL in grades 4 to 9 [26].

### 5.5. Applications for Educational Policy and Practice

The current findings support the need to recognize that there are multiple, evidence-based approaches to defining SLDs in general and SLDs-WL in particular. Given the well documented genetic heterogeneity in oral language, reading, and writing skills and brain differences among contrasting SLDs, it should be common sense that flexible approaches to definition, identification, and differential diagnosis will be needed so that the educational needs of all students can be met in school settings [23] [28]. Assessing cognitive abilities is relevant to meeting the needs of some individuals with SLDs-WL, even if there is no evidence that simply subtracting an achievement score from a cognitive ability score is sufficient for identifying an SLD-WL.

Nor is there evidence that assessing achievement alone is sufficient. There are students who are twice exceptional—both gifted and learning disabled with an SLD [58]. A twice exceptional child may appear to be achieving adequately because achievement scores fall in the average range; but because the giftedness masks the disability or the disability masks the giftedness, either the disability or the giftedness or both may be missed [34] [37]. As a result, the student may not receive appropriate education for either the disability or giftedness. Twice exceptional students are sometimes mistakenly perceived to be either lazy or underachievers; and their learning disabilities may be severe enough to be diagnosed, but the giftedness may never be recognized [34].

Overall, the current results support the use of normed measures of cognitive ability, achievement, and working memory endophenotypes as part of comprehensive evidence-based evaluations in assessing possible twice exceptional status [3]. Importantly, **a**ssessment results should be used not only to identify SLDs and associated patterns of strengths and weaknesses but also to plan, implement, and monitor progress for differentiated instructional strategies for students with SLDs-WL. Differentiated instruction should be designed for both talents/strengths and disabilities as well as cultural and linguistic differences that can occur in students in general as well as those with various exceptionalities. An inclusive mainstream classroom setting is desirable for differentiated instruction so students with SLDs-WL are exposed to the same curriculum as peers [59] [60]. Instructional programs in inclusive settings should also take into account the social emotional needs of students with SLDs. The twice exceptional have lower self-esteem than gifted students without SLDs [61]. Students with SLDs-WL, of average to very superior verbal abilities, may also experience more internalizing and externalizing disorders than students without SLDs-WL [62]. One way to deal with social emotional issues related to learning differences is through bibliotherapy. Students can read about characters experiencing issues similar to what they are experiencing. For resources for helping deal with social emotional issues the twice see [63] [64].

### 5.6. Limitations and Future Directions

The current study was conducted in a research setting with some ethnic and cultural diversity but not as much as many schools in United States or other countries may have in their classrooms. Future research on learning profiles and phenotype profiles should be conducted in school settings with more diverse learners and across countries. The current study was restricted to biologically based SLDs-WL, but future research might also investigate math and other disabilities that do and do not co-occur with SLDs-WL [65] and develop measures for assessing RTI for both talents and SLDs.

Much work remains to achieve the desired goal of FAPE for ALL. To begin with, there are many ways to be exceptional, which may not fit the conventional categories used by schools to classify students by eligibility criteria for receipt of special education services or placement in gifted programs. At the same time numerous controversies continue about how to define eligibility criteria for programs for **specific learning disabilities** (SLDs) [1] [4] [19] [66]**, giftedness** [3] [7] [67], and **“twice” exceptional—both gifted and learning disabled** [34] [68] [69] [70].

Although twice exceptional students vary in the nature of their giftedness and disability [71], the current study focuses on just one of kind of giftedness (verbal—translation of cognitions into oral language) and one kind of disability— persisting specific learning disabilities in written language (SLDs-WL)—in students in grades 4 to 9 (upper elementary, middle school, and transition to high school in the United States where the study was conducted). More research is needed on the variety of ways in which individual students may exhibit specific learning disabilities and giftedness. The current study was also limited by the relatively small sample size even though the sample was carefully ascertained to meet the criteria for persisting SLDs or no history of ever having had SLDs. Future research should evaluate if the current findings replicate in other samples in other settings.

### 5.7. Concluding Remarks

Research that designs and evaluates instruction to facilitate strengths and talents for students in general is emerging [72], but needs to continue to expand to different kinds of abilities and disabilities and the configurations in which they occur. At the same time, school policy needs to evolve to allow educators professional autonomy for flexible translation and implementation of the research into practice rather than pressuring school professionals to rely on rigid implementation of legal policy written by non-educators [73]. It is ironic that it has been assumed there may be a single way to define specific learning disabilities (a plural word) which co-occur in individuals with multiple kinds of abilities and strengths and weaknesses that all vary along continuous distributions. Both science and practice would benefit from redirecting attention from the search for a one size fits all definition of SLDs to creation of evidence-based multi-dimensional conceptual frameworks for defining research variables and assessing and teaching students who exhibit considerable normal variation and heterogeneity in how that variation may sometimes fall outside the normal range.

## Figures and Tables

**Table 1.** Replication study comparing verbally gifted with SLDs-WL and average with SLDs-WL.

Measures	Verbally *Gifted* with SLDs (G-SLDs) *n* = 27		Verbally *Average* with SLDs (Avg-SLDs) *n* = 22		G-SLDs vs. Avg-SLDs	

	*M*	(*SD*)	*M*	(*SD*)	*t*(47)	*d*
*Verbal Compreh* WISC-IV VCI	126.7	(5.7)	99.9	(10.0)	8.74^***^	3.37
*Learning Profiles Oral Language* CELF-4 Form Sent	11.9	(2.4)	9.9	(2.6)	3.14^**^	0.81
WJ-3 Oral Comp	118.0	(7.6)	107.2	(12.5)	3.88^***^	1.07
*Writing Skills* DASH Copy Best	9.7	(2.5)	8.5	(2.2)	2.57^*^	0.52
DASH Copy Fast	8.4	(2.6)	5.9	(2.8)	3.48^***^	0.94
*Reading Skills* WJ-3 Word ID	106.5	(12.2)	95.1	(12.4)	2.66^*^	0.93
WJ-3 Word Attack	98.5	(10.4)	95.0	(9.8)	1.62ns	0.34
TOWRE SWE	102.7	(17.2)	96.4	(17.0)	2.26^*^	0.37
TOWRE PDE	98.8	(20.1)	92.5	(18.2)	1.60ns	0.33
WJ-3 Psg Comp	106.0	(9.0)	93.4	(9.6)	4.17^***^	1.36
*Phenotype Profiles* CTOPP NWR	9.6	(2.1)	9.1	(2.6)	1.05ns	0.24
TOSWRF	97.9	(11.5)	89.3	(11.2)	3.27^**^	0.75
Comes From	0.4	(0.3)	−0.2	(0.7)	0.99ns	1.17
RAN	98.6	(14.7)	105.1	(12.7)	−0.73ns	−.47
Alphabet 15	−1.0	(0.9)	−1.5	(0.7)	1.87ns	0.56
DKEFS Inhibition	9.9	(2.6)	9.2	(3.3)	1.79ns	0.25
RAS	98.8	(11.2)	105.4	(9.8)	−0.48ns	−0.62

*Notes*: ns = not significant.

* *p* < 0.05

** *p* < 0.01

*** *p* < 0.001.

Cohen’s *d* = difference between group means divided by pooled groups’ standard deviation.

**Table 2.** Group comparisons with verbal ability held constant and presence or absence of diagnosed SLDs-WL varying.

Measures	Verbally *Gifted* with SLDs (G-SLDs) *n* = 27		Verbally *Gifted* with no SLDs (G-no SLDs) *n* = 14		Verbally *Average* with SLDs (Avg-SLDs) *n* = 22		Verbally *Average* no SLDs (Avg-no SLDs) *n* = 6		Comparison 1 G-SLDs vs. G-no SLDs		Comparison 2 Avg-SLDs vs. Avg-no SLDs	

	*M*	(*SD*)	*M*	(*SD*)	*M*	(*SD*)	*M*	(*SD*)	*t*(39)	*d*	*t*(26)	*d*
*Verbal Ability* WISC-IV VCI	126.7	(5.7)	128.4	(12.3)	99.9	(10.0)	95.4	(16.3)	−0.02ns	−.20	−0.97ns	−.39
*Learning Profiles Oral Language*^a^ CELF-4 Form Sent	11.9	(2.4)	13.8	(1.7)	9.9	(2.6)	10.1	(9.5)	−2.30^*^	−.91	0.24	.06
WJ-3 Oral Comp	118.0	(7.6)	121.7	(11.1)	107.2	(12.5)	104.0	(2.8)	−0.10ns	−.41	−0.89ns	−.28
*Writing Skills*^b^ DASH Copy Best	9.7	(2.5)	13.7	(2.8)	8.5	(2.2)	15.0	(2.8)	−3.27^**^	−1.51	4.39^***^	2.80
DASH Copy Fast	8.4	(2.6)	12.7	(3.5)	5.9	(2.8)	12.5	(2.1)	−3.07^***^	−1.49	6.57^***^	2.49
*Reading Skills* WJ-3 Word ID^c^	106.5	(12.2)	120.8	(7.0)	95.1	(12.4)	108.5	(17.7)	−3.16^**^	−1.33	1.10ns	.99
WJ-3Word Attack^c^	98.5	(10.4)	107.2	(12.6)	95.0	(9.8)	105.0	(18.4)	−2.60^*^	−.78	1.82ns	.84
TOWRE SWE^c^	102.7	(17.2)	115.0	(14.0)	96.4	(17.0)	111.5	(12.0)	−1.95ns	−.76	2.11^*^	.94
TOWRE PDE^c^	98.8	(20.1)	122.3	(12.2)	92.5	(18.2)	120.5	(6.4)	−3.10^**^	−1.32	2.43^*^	1.69
WJ-3 Psg Comp^a^	106.0	(9.0)	112.8	(15.5)	93.4	(9.6)	100.5	(7.8)	−1.97^§^	−.59	1.33ns	.77
*Phenotype Profiles* CTOPP NWR	9.6	(2.1)	9.7	(3.4)	9.1	(2.6)	10.5	(7.8)	−0.56ns	−.01	0.52ns	.35
TOSWRF	97.9	(11.5)	117.0	(11.2)	89.3	(11.2)	98.5	(5.0)	−2.60^*^	−1.68	2.42^*^	.89
Comes From	0.4	(0.3)	0.8	(0.3)	−0.2	(0.7)	0.1	(0.9)	−3.20^**^	−1.28	0.71ns	.43
RAN	98.6	(14.7)	107.8	(12.0)	105.1	(12.7)	111.5	(3.5)	−1.32ns	−.67	0.89ns	.56
Alphabet 15	−1.0	(0.9)	−0.1	(0.6)	−1.5	(0.7)	−0.1	(0.2)	−3.30^**^	−1.13	4.64^***^	2.08
DKEFS Inhibition	9.9	(2.6)	10.8	(2.1)	9.2	(3.3)	10.5	(2.1)	−1.47ns	−.37	1.40ns	.42
RAS	98.8	(11.2)	109.0	(11.2)	105.4	(9.8)	117.5	(3.5)	−2.10^*^	−.91	1.97^§^	1.35

*Note*:

a Syntax

b Subword

c Word ns = nonsignificant.

§ *p* < 0.10 (trend)

* *p* < 0.05

** *p* < 0.01

*** *p* < 0.001.

Cohen’s *d* = difference between group means divided by pooled groups’ standard deviation.

**Table 3.** Comparing common and unique findings across the two comparisons of contrasting groups.

Measures	Comparison1 G-no SLDs *higher* than G-SLDs	Comparison 2 Avg-no SLDs *higher* than Avg-SLDs
*Learning Profiles Oral Language* CELF-4 Form Sent		
WJ-3 Oral Comp		
*Writing Skills* DASH Copy Best	sig^b^	sig^b^
DASH Copy Fast	sig^b^	sig^b^
Reading Skills WJ-3 Word ID	sig^a^	
WJ-3 Word Attack	sig^a^	
TOWRE SWE		sig^a^
TOWRE PDE		sig^a^
WJ-3 Psg Comp	trend^§^	
*Phenotype Profiles* CTOPP NWR TOSWRF	sig^b^	sig^b^
Comes From	sig^a^	
RAN Alphabet 15	sig^b^	sig^b^
DKEFS Inhibition RAS		

*Note*:

a Indicates that the two comparisons yielded different results for the skills or phenotypes on which the groups were compared; that is, the range of verbal ability in the sample mattered.

b Indicates that the two comparisons yielded the same results for the skills or phenotypes on which the groups were compared; that is, the range of verbal ability in the sample did not matter

§ Trend toward significance in one group only.

## References

[1] Fletcher JM, Coulter WA, Reschly DJ and Vaughn S (2004) Alternative Approaches to the Definition and Identification of Learning Disabilities: Some Questions and Answers. Annals of Dyslexia, 54, 304–331. 10.1007/s11881-004-0015-y 15741940

[2] Sotelo-Dynega M, Flanagan DP and Alfonso VC (2011) Overview of Specific Learning Disabilities In: Flanagan DP and Alfonso VC, Eds., Essentials of Specific Learning Disability Identification, John Wiley and Sons Inc., Hoboken, 203–233.

[3] Pfeiffer SI (2015) Essentials of Gifted Assessment. John Wiley & Sons, Hoboken.

[4] Silliman ER and Berninger VW (2011) Cross-Disciplinary Dialogue about the Nature of Oral and Written Language Problems in the Context of Developmental, Academic, and Phenotypic Profiles. Topics in Language Disorders, 31, 6–23. 10.1097/TLD.0b013e31820a0b5b

[5] Ferri BA, Gregg N and Heggoy SJ (1997) Profiles of College Students Demonstrating Learning Disabilities with and without Giftedness. Journal of Learning Disabilities, 30, 552–559. 10.1177/002221949703000511 9293237

[6] Van Viersen S, Kroesbergen E, Slot E and de Bree E (2016) High Reading Skills Mask Dyslexia in Gifted Children., Journal of Learning Disabilities, 49, 189–199. 10.1177/0022219414538517 24935885

[7] Kaufman SB and Sternberg RJ (2008) Conceptions of Giftedness In: Pfeiffer SI, Ed., Giftedness in Children: Psychoeducational Theory, Research, and Best Practices, Springer, Tallahassee, 71–91. 10.1007/978-0-387-74401-8_5

[8] Ford DY and Whiting GW (2008) Recrui ting and Retaining Underrepresented Gifted Students In: Pfeiffer SI, Ed, Handbook of Giftedness in children: Psychoeducational Theory, Research, and Best Practices, Springer, Tallahassee, 293–308. 10.1007/978-0-387-74401-8_15

[9] Naglieri JA and Ford DY (2003) Addressing Underrepresentation of Gifted Minority Children Using the Naglieri Nonverbal Ability Test (NNAT). Gifted Child Quarterly, 47, 155–160. 10.1177/001698620304700206

[10] Pfeiffer SI, Petscher Y and Jarosewich T (2007) Sharpening Identification Tools. Roeper Review, 29, 206–211. 10.1080/02783190709554410 26346963 PMC4557809

[11] Sternberg RJ (2010) Assessment of Gifted Students for Identification Purposes: New Techniques for a New Millennium. Learning and Individual Differences, 20, 327–336.

[12] Renzulli JS (2004) Identification of Students for Gifted and Talented Programs. Corwin Press, Thousand Oaks.

[13] Robinson N (2008) The Social World of Gifted Children and Youth In: Pfeiffer SI, Ed, Handbook of Giftedness in Children: Psychoeducational Theory, Research, and Best Practices, Springer, Tallahassee, 33–52. 10.1007/978-0-387-74401-8_3

[14] Peterson J (2013) Gender Differences in Identification of Gifted Youth and in Gifted Program Participation: A Meta-Analysis. Contemporary Educational Review, 38, 324–348.

[15] McBee MT (2006) A Descriptive Analysis of Referral Sources for Gifted Identification Screening by Race and Socioeconomic Status. The Journal of Secondary Gifted Education, 18, 103–111.

[16] Bradley R, Danielson L and Doolittle J (2007) Responsiveness to Interventions in Reading: 1997–2007. Teaching Exceptional Children, 39, 8–12. 10.1177/004005990703900502

[17] Gilman B, Lovecky D, Kearney K, Peters D, Wasserman J, Kreger L, (2013) Critical Issues in the Identification of Gifted Students with Co-Existing Disabilities: The Twice Exceptional. Sage Open. 10.1177/2158244013505855

[18] Warner TD, Dede DE, Garvan CW and Conway TW (2002) One Size Does Not Fit All in Specific Learning Disability Assessment across Ethnic Groups. Journal of Learning Disabilities, 35, 500–508. 10.1177/00222194020350060201 15493248

[19] Flanagan DP, Ortiz SO, Alfonso VC and Dynda AM (2006) Integration of Response to Intervention and Norm-Referenced Tests in Learning Disability Identification: Learning from the Tower of Babel. Psychology in the Schools, 43, 807–825. 10.1002/pits.20190

[20] Hale JB, Kaufman A, Naglieri J and Kavale K (2006) Implementation of IDEA: Integrating Response to Intervention and Cognitive Assessment Methods. Psychology in the Schools, 43, 753–770. 10.1002/pits.20186

[21] Wodrich DL, Spencer MLS and Daley KB (2006) Combining RTI & Psychoeducational Assessment: What We Must Assume to Do Otherwise. Psychology in the Schools, 43, 797–825. 10.1002/pits.20189

[22] Schultz EK, Simpson CG and Lynch S (2012) Specific Learning Disability Identification: What Constitutes a Pattern of Strengths and Weaknesses? Learning Disabilities: A Multidisciplinary Journal, 18, 87–97.

[23] Berninger VW, Richards T and Abbott RD (2015) Differential Diagnosis of Dysgraphia, Dyslexia, and OWL LD: Behavioral and Neuroimaging Evidence. Reading and Writing: An Interdisciplinary Journal, 28, 1119–1153. 10.1007/s11145-015-9565-0 PMC4553247 26336330

[24] Berninger VW and Abbott RD (2013) Differences between Children with Dyslexia Who Are and Are Not Gifted in Verbal Reasoning. Gifted Child Quarterly, 57, 223–233. 10.1177/0016986213500342 PMC3829472 24249873

[25] Niedo J, Abbott RD and Berninger VW (2014) Predicting Levels of Reading and Writing Achievement in Typically Developing, English-Speaking 2nd and 5th Graders. Learning and Individual Differences, 32, 54–68. 24948868 10.1016/j.lindif.2014.03.013 PMC4058427

[26] Sanders EA, Berninger VW and Abbott RD (2017) Sequential Prediction of Literacy Achievement for Specific Learning Disabilities Contrasting in Impaired Levels of Language in Grades 4 to 9. Journal of Learning Disabilities. 10.1177/0022219417691048 PMC5538955 28199175

[27] Katusic SK, Colligan RC, Weaver AL and Barbaresi WJ (2009) The Forgotten Learning Disability—Epidemiology of Written Language Disorder in a Population-Based Birth Cohort (1976–1982), Rochester, Minnesota. Pediatrics, 123, 1306–1313. 10.1542/peds.2008-2098 19403496 PMC2923476

[28] Berninger VW (2015) Interdisciplinary Frameworks for Schools: Best Professional Practices for Serving the Needs of All Students. American Psychological Association, Washington DC. 10.1037/14437-002

[29] Greenblatt E, Mattis S and Trad P (1990) Nature and Prevalence of Learning Disabilities in a Child Psychiatric Population. Developmental Neuropsychology, 6, 71–83. 10.1080/87565649009540451

[30] Vellutino F, Scanlon D and Tanzman M (1991). Bridging the Gap between Cognitive and Neuropsychological Conceptualizations of Reading Disabilities. Learning and Individual Differences, 3, 181–203.

[31] Stahl S and Nagy W (2005) Teaching Word Meaning. Erlbaum, Malwah.

[32] American Psychiatric Association (1994) Diagnostic and Statistical Manual of Mental Disorders. 4th Edition, Author, Washington DC.

[33] Lovett BJ and Lewandowski LJ (2006) Gifted Students with Learning Disabilities: Who Are They? Journal of Learning Disabilities, 39, 515–527. 10.1177/00222194060390060401 17165619

[34] Brody LE and Mills C (1997) Gifted Children with Learning Disabilities: A Review of the Issues. Journal of Learning Disabilities, 30, 282–297. 10.1177/002221949703000304 9146095

[35] McKenzie RG (2010) The Insufficiency of Response to Intervention in Identifying Gifted Students with Learning Disabilities. Learning Disabilities Research & Practice, 25, 161–168. 10.1111/j.1540-5826.2010.00312.x

[36] Ruban LM and Reis SM (2005) Identification and Assessment of Gifted Students with Learning Disabilities. Theory into Practice, 44, 115–124. 10.1207/s15430421tip4402_6

[37] Assouline S, Foley-Nicpon M and Huber D (2006) The Impact of Vulnerabilities and Strengths on the Academic Experiences of Twice-Exceptional Students: A Message to School Counselors. BioMedSearch, 10 10.5330/prsc.10.1.y0677616t5j15511

[38] Berninger V and Richards T (2010). Inter-Relationships among Behavioral Markers, Genes, Brain, and Treatment in Dyslexia and Dysgraphia. Future Neurology, 5, 597–617. 10.2217/fnl.10.22 20953351 PMC2953808

[39] Wagner R and Torgesen J (1987) The Nature of Phonological Processing and Its Causal Role in the Acquisition of Reading Skills. Psychological Bulletin, 101, 192–212. 10.1037/0033-2909.101.2.192

[40] Berninger VW (2009) Highlights of Programmatic, Interdisciplinary Research on Writing. Learning Disabilities. Research and Practice, 24, 68–79. 10.1111/j.1540-5826.2009.00281.x PMC2717633 19644563

[41] Mather N and Wendling BJ (2011) How SLD Manifests in Writing In: Flanagan DP and Alfonso VC, Eds., Essentials of Specific Learning Disability Identification, John Wiley and Sons, Inc., Hoboken, 65–88.

[42] Nagy W, Berninger V and Abbott R (2006) Contributions of Morphology beyond Phonology to Literacy Outcomes of Upper Elementary and Middle School Students. Journal of Educational Psychology, 98, 134–147. 10.1037/0022-0663.98.1.134

[43] Baddeley A, Gathercole S and Papagno C (1998) The Phonological Loop as a Language Learning Device. Psychological Review, 105, 158–173. 10.1037/0033-295X.105.1.158 9450375

[44] Swanson HL (2006) Working Memory and Learning Disabilities: Both Phonological and Executive Processing Deficits Are Important In: Alloway TP and Gathercole SE, Eds., Working Memory and Neurodevelopmental Disorders, Psychology Press, London, 59–88.

[45] Delis DC, Kaplan E and Kramer JH (2001) Delis-Kaplan Executive Function System (DKEFS). Psychological Corporation, San Antonio.

[46] Swanson HL (1993) Executive Processing in Learning Disabled Readers. Intelligence, 17, 117–149.

[47] Wolf M (1986) Rapid Alternating Stimulus Naming in the Developmental Dyslexias. Brain and Language, 27, 360–379. 3513900 10.1016/0093-934x(86)90025-8

[48] Swanson L and Siegel L (2001) Learning Disabilities as a Working Memory Deficit. Issues in Education, 7, 1–48.

[49] Wechsler D (2003) Wechsler Intelligence Scale for Children. 4th Edition, WISC-IV, The Psychological Corporation, San Antonio.

[50] Woodcock R and Johnson ME (2001) Tests of Cognitive Abilities III. Riverside Publishing, Itasca.

[51] Semel E, Wiig EH and Secord WA (2003) Clinical Evaluations of Language Fundamentals 4th Edition: Examiner’s Manual. Harcourt Assessment Inc., San Antonio.

[52] Barnett A, Henderson L, Scheib B and Schulz C (2007) Detailed Assessment of Speed of Handwriting (DASH) Copy Best and Fast. Pearson, London.

[53] Woodcock R, McGrew K and Mather N (2001) Woodcock-Johnson III Psychoeducational Test Battery. Riverside, Itasca.

[54] Torgesen JK, Wagner RK and Rashotte CA (1999) Test of Word Reading Efficiency (TOWRE). PRO-ED, Austin.

[55] Wagner R, Torgesen J and Rashotte C (1999) The Comprehensive Test of Phonological Processing (CTOPP). PRO-ED, Austin.

[56] Mather N, Roberts R, Hammill D and Allen E (2008) Tests of Silent Word Reading Fluency TOSWRF. Pro-Ed, Austin.

[57] Wolf M and Denckla M (2003) RAN/RAS Rapid Automatized Naming and Rapid Alternating Stimulus Tests. Pro-Ed, Austin.

[58] Nielsen ME and Higgins LD (2005) The Eye of the Storm: Services and Programs for Twice-Exceptional Learners. Teaching Exceptional Children, 38, 8–15.

[59] Banerji M and Dailey RA (1995) A Study of the Effects of an Inclusion Model on Students with Specific Learning Disabilities. Journal of Learning Disabilities, 28, 511–531. 10.1177/002221949502800806 7595042

[60] Tomlinson CA (2014) The Differentiated Classroom: Responding to the Needs of All Learners. 2nd Edition, ASCD, Alexandria.

[61] Foley-Nicpon M and Assouline S (2015) Counseling Considerations for the Twice-Exceptional Client. Journal of Counseling & Development, 93, 202–211. 10.1002/j.1556-6676.2015.00196.x

[62] Nielsen K, Haberman K, Richards T, Abbott R, Mickail T and Berninger V (in press) Emotional and Behavioral Correlates of Persisting Specific Learning Disabilities in Written Language (SLDs-WL) during Middle Childhood and Early Adolescence. Journal of Psychoeducational Assessment. 10.1177/0734282917698056 PMC6291223 30555207

[63] Halstead JW (2009) Some of My Best Friends are Books: Guiding Gifted Readers. 3rd Edition, Great Potential Press, Scottsdale.

[64] King EW (2005) Resources on Twice-Exceptional Students. Teaching Exceptional Children, 38, 55–56.

[65] Berninger VW (2011) Evidence-Based Differential Diagnosis and Treatment of Reading Disabilities with and without Comorbidities in Oral Language, Writing, and Math: Prevention, Problem-Solving Consultation, and Specialized Instruction In: Flanagan DP and Alfonso VC, Eds., Essentials of Specific Learning Disability Identification, John Wiley and Sons Inc., Hoboken, 203–233.

[66] Fletcher JM, Denton C and Francis DJ (2005) Validity of Alternative Approaches for the Identification of Learning Disabilities: Operationalizing Unexpected Underachievement. Journal of Learning Disabilities, 38, 545–552. 10.1177/00222194050380061101 16392697

[67] Renzulli JS (1978) What Makes Giftedness? Reexamining a Definition. Phi Delta Kappan, 60, 180–184.

[68] Assouline SG, Foley-Nicpon MF and Whiteman C (2010) Cognitive and Psychosocial Characteristics of Gifted Students with Written Language Disability. Gifted Child Quarterly, 54, 101–115. 10.1177/0016986209355974

[69] Foley-Nicpon M, Allmon A, Sieck B and Stinson R (2011) Empirical Investigation of Twice-Exceptionality: Where Have We Been and Where Are We Going? Gifted Child Quarterly, 55, 3–17. 10.1177/0016986210382575

[70] Winebrenner S (2003) Teaching Strategies for Twice-Exceptional Students. Intervention in School and Clinic, 38, 131–137. 10.1177/10534512030380030101

[71] Ronksley-Pavia M (2015) A Model of Twice-Exceptionality: Explaining and Defining the Apparent Paradoxical Combination of Disability and Giftedness in Childhood. Journal for the Education of the Gifted, 38, 318–340. 10.1177/0162353215592499

[72] Baum SM, Schader RM and Hebert TP (2014) Through a Different Lens: Reflecting on a Strength-Based, Talent-Focused Approach for Twice-Exceptional Learners. Gifted Child Quarterly, 58, 311–327. 10.1177/0016986214547632

[73] Volker MA, Lopata C and Cook-Cottone C (2006) Assessment of Children with Intellectual Giftedness and Reading Disabilities. Psychology in the Schools, 43, 855–869. 10.1002/pits.20193

